# Vitamin (B1, B6, B12, D, and Folate) and Mineral (Iron, Selenium, and Magnesium) Status Across the Spectrum of Severe Obesity: An Integrative Multivariate Analysis of Metabolic and Nutritional Profiles

**DOI:** 10.3390/nu18172938

**Published:** 2026-09-07

**Authors:** Hannah Sabados, Sandra Möwius, Ute M. Stern, Maurice Michel, Jörn M. Schattenberg, Verena Keller

**Affiliations:** 1 Nutrition and Dietetics, Saarland University, 66421 Homburg, Germanysandra.moewius@uni-saarland.de (S.M.); 2 Department of Internal Medicine II, Saarland University, 66421 Homburg, Germany; ute.stern@uks.eu (U.M.S.); maurice.michel@uks.eu (M.M.); joern.schattenberg@uks.eu (J.M.S.); 3 Faculty of Medicine, Saarland University, 66123 Saarbrücken, Germany

**Keywords:** obesity, micronutrients, vitamins, metabolic dysfunction, liver steatosis, type 2 diabetes, nutritional assessment, machine learning

## Abstract

**Background:** Obesity is frequently accompanied by metabolic dysfunction and micronutrient deficiencies, yet data on individuals with severe obesity (BMI ≥ 50 kg/m^2^) remain limited. Understanding how vitamin and mineral status changes across the adiposity continuum may optimize personalized nutritional management in severe obesity. **Methods:** We analyzed anthropometric, metabolic, hepatic, and micronutrient parameters in a retrospective, single-center cohort of adults evaluated before bariatric surgery, with a BMI range of 30 to 91 kg/m^2^ (mean 48.7 ± 8.7). Measurements included bioimpedance-derived body composition, liver elastography (CAP and stiffness), and fasting laboratory parameters, vitamins B1, B6, B12, D, and folate and minerals (iron, selenium, and magnesium). Correlation analysis, principal component analysis (PCA), and random forest (RF) classification were applied to identify the variables most strongly associated with BMI and metabolic phenotypes. **Results:** Several micronutrient concentrations, most notably vitamin D, folate and vitamin B6, declined progressively with increasing BMI. Vitamin D additionally correlated inversely with HbA1c. PCA revealed a separation of participants with extreme obesity driven by anthropometric and hepatic parameters, while micronutrients clustered inversely. RF classification identified HbA1c, hepatic enzymes (ALAT and γ-GT) and body-composition measures (waist circumference and visceral fat) as the strongest discriminators of T2DM, MASLD and high BMI, respectively. Combined MASLD and T2DM was associated with the most pronounced vitamin B6 depletion, whereas vitamin D was low across metabolic subgroups. **Conclusions:** Severe obesity is associated with distinct micronutrient depletion patterns that mirror metabolic deterioration. Integrating nutritional and metabolic profiling enables refined risk stratification and may, pending prospective validation, inform targeted supplementation strategies in individuals with severe obesity.

## 1. Introduction

Obesity is a major global health challenge and is closely linked to the development of multiple chronic diseases, including type 2 diabetes, cardiovascular disease and metabolic-associated fatty liver disease (MASLD) [1,2,3] that has recently been reclassified under a multisociety nomenclature [4]. Also, breast, gastric and other cancers are associated with obesity [5,6,7,8,9], as summarized by the IARC Working Group [10]. Excess adiposity contributes to substantial morbidity, mortality, and economic burden worldwide [11,12], with global obesity prevalence rising over recent decades and higher BMI linked to increased all-cause mortality [13,14]. In the state of severe or class III obesity, the risk of related complications is especially pronounced, with visceral fat, ectopic lipid deposition, and adipose tissue dysfunction driving metabolic dysregulation [15,16,17,18].

At the same time, vitamins and other micronutrients play critical roles in energy metabolism, hormone regulation, inflammation control [19,20,21], and immune and endocrine function [22]. Deficiencies or altered metabolism of vitamins have been increasingly recognized in obesity and its metabolic complications. For example, vitamin D deficiency is common in individuals with obesity [23], its assessment and supplementation are addressed in recent clinical practice guidelines [24], and dietary vitamin status has been linked to obesity-related metabolic outcomes. Moreover, associations between vitamin B status and adiposity have been observed, with several studies reporting lower circulating vitamin B6 [25] and B12 [26,27] levels in overweight or obese individuals. Recent clinical and systems-level studies have expanded these observations. A large cross-sectional study identified obesity, hypertension, and hypertriglyceridemia as key risk factors for type 2 diabetes, reinforcing the strong interconnection between metabolic risk and adiposity [28]. In line with this, transcriptomic and meta-analytic data demonstrated that vitamin D supplementation enhances the effectiveness of exercise-induced weight loss, especially in older or obese adults, through improved skeletal muscle metabolic responsiveness [29]. Complementary evidence from a UK Biobank analysis found that low serum 25(OH)D and high BMI synergistically accelerate biological aging, suggesting that vitamin D deficiency may amplify metabolic stress in obesity [30]. Further studies have highlighted that interventions modifying nutrient absorption or gut microbiota, such as probiotic or synbiotic supplementation after bariatric surgery, can improve BMI, HbA1c, and vitamin D status, supporting a bidirectional link between micronutrient levels and metabolic adaptation [31]. Vitamin D supplementation itself has been shown to exert broad benefits across clinical contexts, from skeletal protection to modulation of autoimmune and metabolic diseases, though its efficacy is influenced by factors such as body weight, absorption capacity, and renal function [32]. Finally, vitamin D deficiency has been associated with higher BMI, HbA1c, and inflammatory markers in both men and women, however to different extents, emphasizing the complex interplay between adiposity, inflammation, and micronutrient homeostasis between sexes [33]. Together, these findings point toward a multifaceted relationship between obesity, metabolic disease, and vitamin status in a sex-dependent manner. Understanding how micronutrient imbalances interact with disease progression in severely obese individuals could help to identify modifiable nutritional and metabolic targets to improve long-term outcomes.

Despite the increasing recognition of vitamin deficiencies in obesity, only a few studies have explicitly addressed micronutrient status in individuals with severe obesity (BMI ≥ 50 kg/m^2^). Often, reports pool all patients with class III obesity (BMI ≥ 40 kg/m^2^) or analyze mixed bariatric cohorts without stratification by BMI severity, and correlates among patients with BMI ≥ 50 kg/m^2^ remain insufficiently characterized. As highlighted by prior bariatric research, super-obese patients (here defined as BMI ≥ 50 kg/m^2^) may differ physiologically and nutritionally from those with lower-grade (class I–II) obesity, requiring distinct clinical and biochemical considerations. Accordingly, a focused analysis of vitamin profiles in this extreme BMI range could reveal metabolic signatures that are masked in more heterogeneous cohorts.

In the present study, we investigated a cohort of patients with severe obesity evaluated prior to bariatric surgery, encompassing a broad spectrum of adiposity levels, with a substantial proportion (39.4%) having a BMI ≥ 50 kg/m^2^. Alongside anthropometric measures such as BMI and waist circumference, we comprehensively assessed metabolic, hepatic, and micronutrient parameters, including circulating levels of vitamins D, B1, B6, B12, and folate. By integrating clinical markers with vitamin profiles, our objective was to determine whether vitamin (B1, B6, B12, D, and folate) and mineral (iron, selenium, and magnesium) status changes across degrees of adiposity—continuously or with a threshold at BMI ≥ 50 kg/m^2^—and how it relates to comorbid metabolic disorders such as type 2 diabetes mellitus (T2DM) and metabolic dysfunction-associated steatotic liver disease (MASLD). This approach allows us to identify patterns of deficiency and disease-specific alterations that may provide insight into the metabolic consequences of extreme obesity and inform individualized nutritional management strategies.

## 2. Materials and Methods

### 2.1. Clinical Sampling

**Figure 1:**
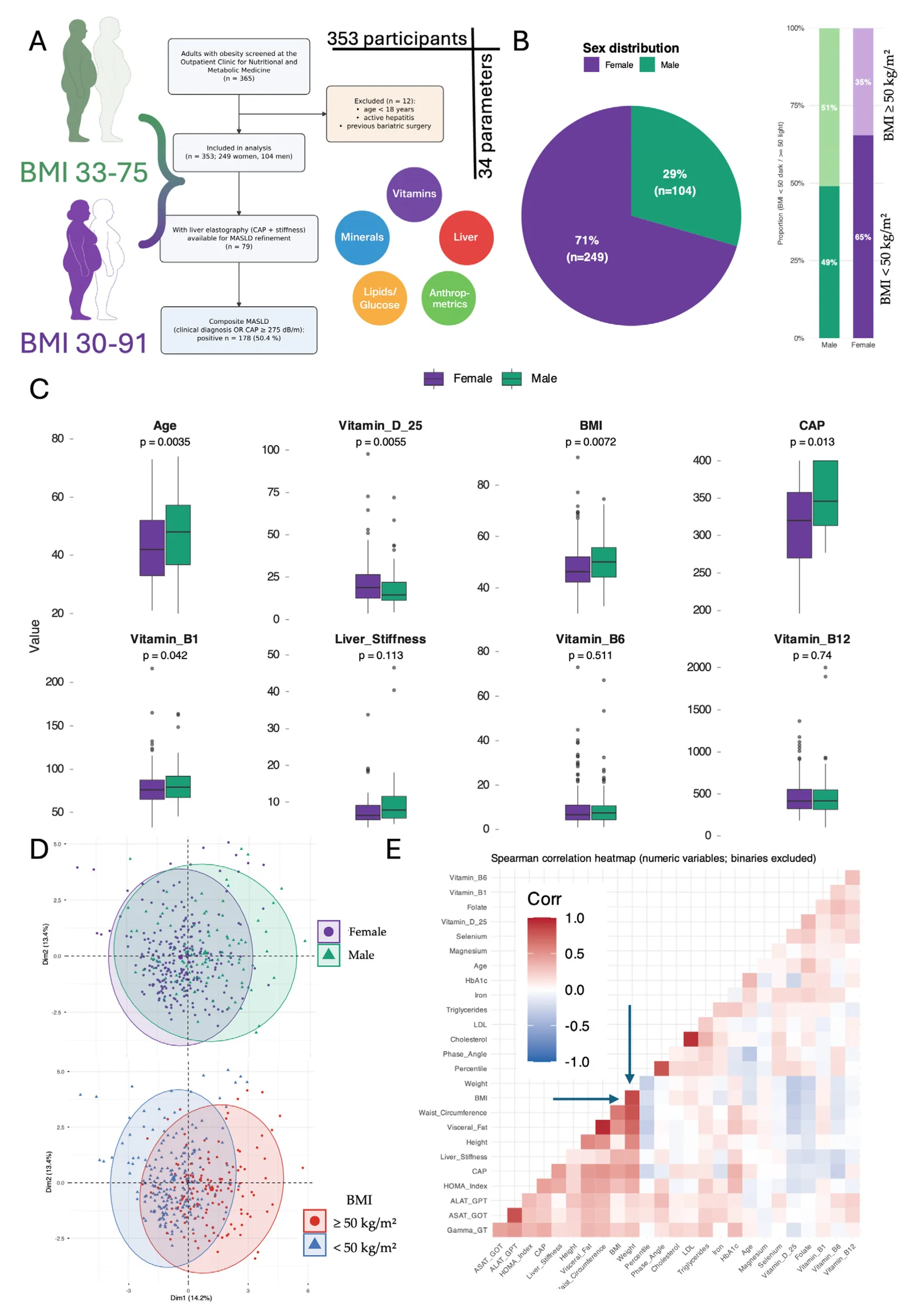
Cohort overview and global data structure and the cohort with exclusions. (**A**) Study design: 353 adults (249 women, 104 men) assessed for 34 clinical and biochemical variables, of which 26 continuous parameters fall into five functional families (vitamins, minerals, liver markers, lipids/glucose, and anthropometrics); BMI ranged from 30 to 91 kg/m^2^ in women and 33 to 75 kg/m^2^ in men. (**B**) Sex distribution (pie chart) and the proportion of participants with high (≥50 kg/m^2^) versus low (<50 kg/m^2^) BMI within each sex. (**C**) Boxplots of selected variables by sex (two-sided Wilcoxon rank-sum test; *p* shown per panel). (**D**) Principal component analysis of all numeric variables (columns with >10% missing values removed, remaining values mean-imputed and z-scaled), individuals colored by sex (top) and by BMI group (cutoff 50 kg/m^2^; bottom); ellipses denote 95% confidence regions. (**E**) Spearman correlation heatmap of the numeric variables with hierarchical clustering (Ward.D2 linkage); arrows exemplarily highlight the correlation between BMI and weight as the anchor of the anthropometric cluster.

Participants were seen at the Outpatient Clinic for Nutritional and Metabolic Medicine of the Department of Internal Medicine, Saarland University Hospital (Homburg, Germany), which focuses on the metabolic evaluation, preventive screening, and dietary counseling of adults with obesity and related metabolic conditions. Eligible participants were aged 18 years or older. Data from all patients attending this program were subsequently analyzed retrospectively in accordance with the principles of the Declaration of Helsinki and under approval of the local ethics committee (approval no. 130/24, date of approval 27 August 2024), with all records processed in pseudonymized form. Of 365 adults screened during the study period, twelve were excluded: five with a BMI below 30 kg/m^2^, three with previous bariatric surgery, two in whom BMI could not be determined because anthropometric data were incomplete, one aged under 18 years at assessment, and one with active hepatitis C. The remaining 353 participants were retained for analysis, of whom 79 additionally underwent liver elastography; the numbers screened and excluded, with reasons, are summarized in the participant flow diagram (Figure 1A). Source records were verified and data curation was kept to the minimum necessary: a few anthropometric transcription errors and implausible laboratory values were corrected or set to missing, and one participant with active hepatitis was excluded; full details are given in the Supplementary Methods. A food-frequency questionnaire was available for 67 participants; because an analysis restricted to these 67 individuals would be statistically underpowered and unstable, dietary-intake data were not further analyzed. Participants without overt metabolic comorbidities apart from elevated BMI were classified as metabolically healthy individuals, allowing inclusion of a broad spectrum of metabolic phenotypes. Clinical and laboratory data were collected at a single time point under standardized conditions following an overnight fast. Inclusion criteria encompassed the availability of core anthropometric data (BMI, age, and sex) and fasting laboratory data including micronutrient measurements, as well as sufficient sample material for biochemical analyses; liver elastography was available in a subset (n = 79), and per-variable missingness is reported in Supplementary Table S1. Participants with type 2 diabetes mellitus (T2DM) or metabolic dysfunction-associated steatotic liver disease (MASLD) were classified according to established diagnostic criteria (fasting glucose ≥ 126 mg/dL or HbA1c ≥ 6.5% for T2DM; a documented clinical diagnosis of hepatic steatosis that, where liver elastography was available, was strengthened by a measured controlled attenuation parameter (CAP) ≥ 275 dB/m—a participant being classified as having MASLD if either criterion was met; and liver stiffness was analyzed separately as a fibrosis marker and did not define MASLD). Because the clinical diagnosis may under-detect steatosis, this composite definition was adopted; in the 79 participants with elastography the measured CAP reclassified 14 clinically unlabeled participants as MASLD-positive, giving a composite MASLD prevalence of 50.4% (178/353) versus 46.5% (164/353) for the clinical diagnosis alone. Analogously, and because a documented clinical diagnosis of type 2 diabetes may under-detect diabetes, T2DM was defined as a composite of the documented clinical diagnosis or a measured HbA1c ≥ 6.5%; this identified 15 participants with an HbA1c in the diabetic range as newly diagnosed T2DM cases, giving a composite T2DM prevalence of 28.9% (102/353). No participant was receiving a GLP-1 receptor agonist at the time of assessment. Exclusion criteria included acute infection or inflammation, pregnancy, active malignancy, known chronic liver diseases of non-metabolic origin (e.g., viral hepatitis, autoimmune or cholestatic liver disease), excessive alcohol consumption, and incomplete datasets preventing reliable analysis. Comprehensive demographic, anthropometric, and laboratory data were recorded for all participants, including measurements of liver function, lipid metabolism, glucose homeostasis, and micronutrient status. For selected analyses, participants were stratified according to sex, presence or absence of MASLD and T2DM, and BMI group (low vs. high, threshold 50 kg/m^2^), to assess metabolic and nutritional patterns across clinically relevant subgroups.

### 2.2. Laboratory Parameters and Measurements

All measurements were conducted as part of a comprehensive nutritional and metabolic assessment in adult participants. Standardized procedures and validated instruments were used to ensure comparability and analytical accuracy across all variables. Anthropometric parameters including body weight, height, and waist circumference were measured with participants wearing light clothing and barefoot. Body composition, including visceral fat and phase angle, was determined by bioelectrical impedance analysis using a calibrated seca mBCA 515 device (seca GmbH, Hamburg, Germany). The body mass index (BMI) was calculated as weight divided by height squared (kg/m^2^). Liver status was evaluated using transient elastography (FibroScan^®^ 530 Compact, Echosens, Paris, France). The controlled attenuation parameter (CAP) quantified hepatic steatosis, while liver stiffness provided a non-invasive estimate of fibrosis. All measurements were carried out by trained operators according to manufacturer specifications. Venous blood samples were collected in the morning following an overnight fast. Serum and plasma were promptly separated and analyzed at the Central Laboratory of the University Hospital, which operates under internal and external quality control standards in accordance with RiliBÄK (Guidelines of the German Medical Association for Quality Assurance in Laboratory Medicine). Routine laboratory parameters including liver enzymes (ASAT/GOT, ALAT/GPT, and γ-GT), total cholesterol, LDL cholesterol, and triglycerides were measured enzymatically on an Abbott Alinity c analyzer (Abbott Diagnostics, Wiesbaden, Germany). HbA1c was determined by high-performance liquid chromatography (HPLC) using an Abbott Alinity hq system. Fasting glucose and insulin were analyzed, and the homeostatic model assessment of insulin resistance (HOMA1-IR) was calculated as (fasting insulin × fasting glucose)/22.5. Micronutrients and vitamins were determined using established, standardized methods. Vitamin D (25(OH)D) and folate were measured by chemiluminescent microparticle immunoassay (CMIA). Vitamins B1, B6, and B12 were analyzed via liquid chromatography coupled to tandem mass spectrometry (LC–MS/MS) to ensure high analytical specificity and sensitivity. Iron, magnesium, and selenium concentrations were quantified by inductively coupled plasma mass spectrometry (ICP–MS). All laboratory analyses were performed by certified medical laboratory scientists using validated protocols and routine calibration. Reproducibility and accuracy were assured through internal controls and participation in external proficiency testing programs.

### 2.3. Computational Analysis

Data preprocessing and harmonization: All analyses were performed in R (version 4.3.2). A full description of the software environment, package versions and analysis parameters is provided in the Supplementary Methods. Full preprocessing details, the assignment of variables to five functional families, and the exact correlation, PCA and random-forest parameters are provided in the Supplementary Methods. In brief, missing values were standardized to NA, binary variables (MASLD, T2DM, and high cholesterol; statin use was documented for only 4 of 353 participants and was therefore not analyzed) were used only for stratified group comparisons and excluded from correlation and PCA analyses, and Spearman correlations between BMI and each numeric variable were computed for the whole cohort and separately by sex. A summary dataset was generated with category annotations and used to create a sex-comparison scatter plot (r_female vs. r_male) and a global ranking of pooled correlations (sex-agnostic), sorted by r magnitude. Correlation matrices among numeric variables were computed using pairwise complete observations and visualized with hierarchical clustering (Ward.D2 linkage). PCA of numeric variables: Principal component analysis (PCA) was performed on numeric variables after controlled preprocessing to handle missing data. Columns with >10% missing values were removed. For the remaining numeric variables, missing entries were imputed by the column mean. All numeric variables were then z-scaled (mean = 0, SD = 1) before PCA. PCA was carried out using FactoMineR::PCA(graph = FALSE) and visualized with factoextra::fviz_pca_ind. Separate color mappings were used for sex and BMI group (cutoff = 50 kg/m^2^, labeled “low BMI” vs. “high BMI”). Ellipses correspond to 95% confidence regions of individuals per group. Group-wise comparisons and distribution analyses: Sex differences in individual biomarkers were evaluated using two-sided Wilcoxon rank-sum tests. For disease stratifications (MASLD and T2DM), group comparisons were performed with Welch’s ANOVA followed by Games–Howell post hoc testing as implemented in the ggstatsplot package. These tests were chosen for robustness against unequal variances and unbalanced group sizes. The −log10(*p*) overview in Figure 3A was instead based on two-sided Wilcoxon rank-sum tests comparing disease-positive and disease-negative participants within each sex. In the Results, group values are reported as the mean followed by the median in parentheses. Vitamin stratification analysis per sub-cohort: To visualize combined influences of sex, MASLD, T2DM, and BMI on vitamin status, participants were grouped into all combinations of Sex × MASLD × T2DM × BMI group (2 × 2 × 2 × 2 = 16 strata). Group-level summaries were computed as the mean of each vitamin (Vitamin_B1, B6, B12, D_25, and Folate) across individuals in that stratum. For visualization, vitamins were z-scored row-wise across groups (each vitamin normalized across its 16 group means). This highlights relative depletion or enrichment per vitamin across strata. Vitamins (rows) were clustered using Euclidean distance of the z-profiles; group columns were ordered by the factor combination of Sex, MASLD, T2DM, and BMI group. Heatmaps were generated with ggplot2::geom_tile using a diverging blue–white–red scale centered at zero. Machine learning classification: To estimate the predictive value of biomarker profiles, random forest classifiers were trained for three binary outcomes: (1) T2DM (Yes/No); (2) high BMI vs. low BMI (cutoff = 50 kg/m^2^); (3) MASLD (Yes/No). Each model used an otherwise identical predictor set, from which the target itself was removed: all numeric variables except BMI, height, weight, total cholesterol and the high-cholesterol indicator (excluded from all three models to avoid trivial or collinear predictors), plus sex and the binary disease indicators. CAP and liver stiffness, which enter the MASLD case definition, were excluded from the MASLD model from the outset. HbA1c, which enters the T2DM case definition, was retained in the primary T2DM model and removed in a sensitivity analysis (Supplementary Figure S3); LDL cholesterol and triglycerides were retained. Rather than discarding incomplete variables, missing values were imputed by the column median and a binary missing-value indicator was added for each variable. Models were trained with 1000 trees using the ranger package as probability forests, and performance was evaluated on out-of-bag predictions. Variable importance was quantified by permutation importance. Performance was evaluated by ROC analysis (pROC), reporting the out-of-bag AUC with DeLong 95% confidence intervals; the shaded confidence bands of the ROC curves were obtained by bootstrap resampling (200 replicates). Comparative AUCs across the three classifiers were summarized in a single bar plot.

We used the following R packages (in R version 4.5.1): tidyverse [34], janitor [35]; ranger [36] for random-forest classification; pROC [37] for ROC-curve and AUC estimation; FactoMineR [38] and factoextra for principal-component analyses; and scales [39] for axis and color control. group-comparison plots used ggstatsplot and break-point analysis used the package segmented [40].

## 3. Results

### 3.1. Increasing Adiposity Is Associated with Hepatic Dysfunction and Declining Micronutrient Status

We analyzed a total of 353 adults (249 females and 104 males) with obesity, covering a wide BMI range from 30 to 91 kg/m^2^ (mean 48.7, median 47.5 kg/m^2^; Figure 1A,B). For each participant, 34 clinical and biochemical variables were recorded, of which 26 continuous parameters were grouped into five functional domains (anthropometric measures, liver function, lipid and glucose metabolism, minerals, and vitamins) and entered the quantitative analyses, while the remaining variables (identifier, sex, smoking status, a binary food-frequency-questionnaire indicator, and the binary disease indicators) served descriptive and stratification purposes (Table 1; detailed baseline characteristics with per-variable missingness are provided in Supplementary Table S1). This integrative design enabled a comprehensive characterization of metabolic status and micronutrient balance. Across the cohort, men displayed significantly higher BMI (mean 50.7 vs. 47.9 kg/m^2^ (median 50.1 vs. 46.3), *p* = 0.0072) and greater hepatic fat content, reflected by increased controlled attenuation parameter (CAP) values (mean 349 vs. 315 dB/m (median 346 vs. 320), *p* = 0.013). Men were also slightly older (mean 47.0 vs. 42.9 years (median 48 vs. 42), *p* = 0.0035) and showed higher vitamin B1 concentrations (mean 82.5 vs. 77.7 (median 79.3 vs. 76.2), *p* = 0.042). Vitamin D, in contrast, was higher in women (mean 21.2 vs. 18.0 (median 18.9 vs. 14.6), *p* = 0.0055), whereas no sex-specific differences were detected for vitamins B6 or B12, while liver stiffness showed a non-significant trend toward higher values in men (*p* = 0.113; Figure 1C). The complete comparison is provided in Supplementary Figure S1, confirming that anthropometric and hepatic parameters most strongly discriminate between sexes.

To obtain a general overview of the dataset structure, we performed a principal component analysis (PCA) across all numeric variables and compared the first two PCs in scatter plots, colored by sex and BMI group (Figure 1D). The resulting distribution revealed a pronounced clustering along BMI- and liver enzyme-related variance, with individuals separating primarily by adiposity level rather than by sex. The first principal component (PC1) was dominated by anthropometric and hepatic markers, with weaker positive contributions of the lipid parameters (leading PC1 loadings: weight 0.78, BMI 0.68, ALAT/GPT 0.52, ASAT/GOT 0.44, γ-GT 0.39; Supplementary Figure S2 and Supplementary Table S2) while micronutrients such as folate and vitamin D contributed inversely to the same axis (vitamin D and folate loading negatively on PC1). This pattern suggests a close alignment of liver function and adiposity with overall metabolic stress, and a progressive decline of micronutrient status with rising steatosis. The second principal component (PC2) received its strongest positive loadings from the transaminases (ASAT/GOT 0.58 and ALAT/GPT 0.54), cholesterol (0.51) and iron (0.45), with further positive loadings from vitamin B12, selenium, folate and vitamin B6 (0.43–0.34), contrasting with negative loadings for body weight and BMI. PC2 thus captures a predominantly micronutrient- and transaminase-related axis opposing anthropometric measures. Together, these two dimensions summarize the metabolic and nutritional heterogeneity of the cohort. To gain further insight into the interdependencies among these variables, we next performed pairwise correlation analyses, which substantiated these relationships (Figure 1E).

### 3.2. Sex-Stratified Correlations Show Concordant BMI Associations and Vitamin Depletion with Increasing Adiposity

**Figure 2:**
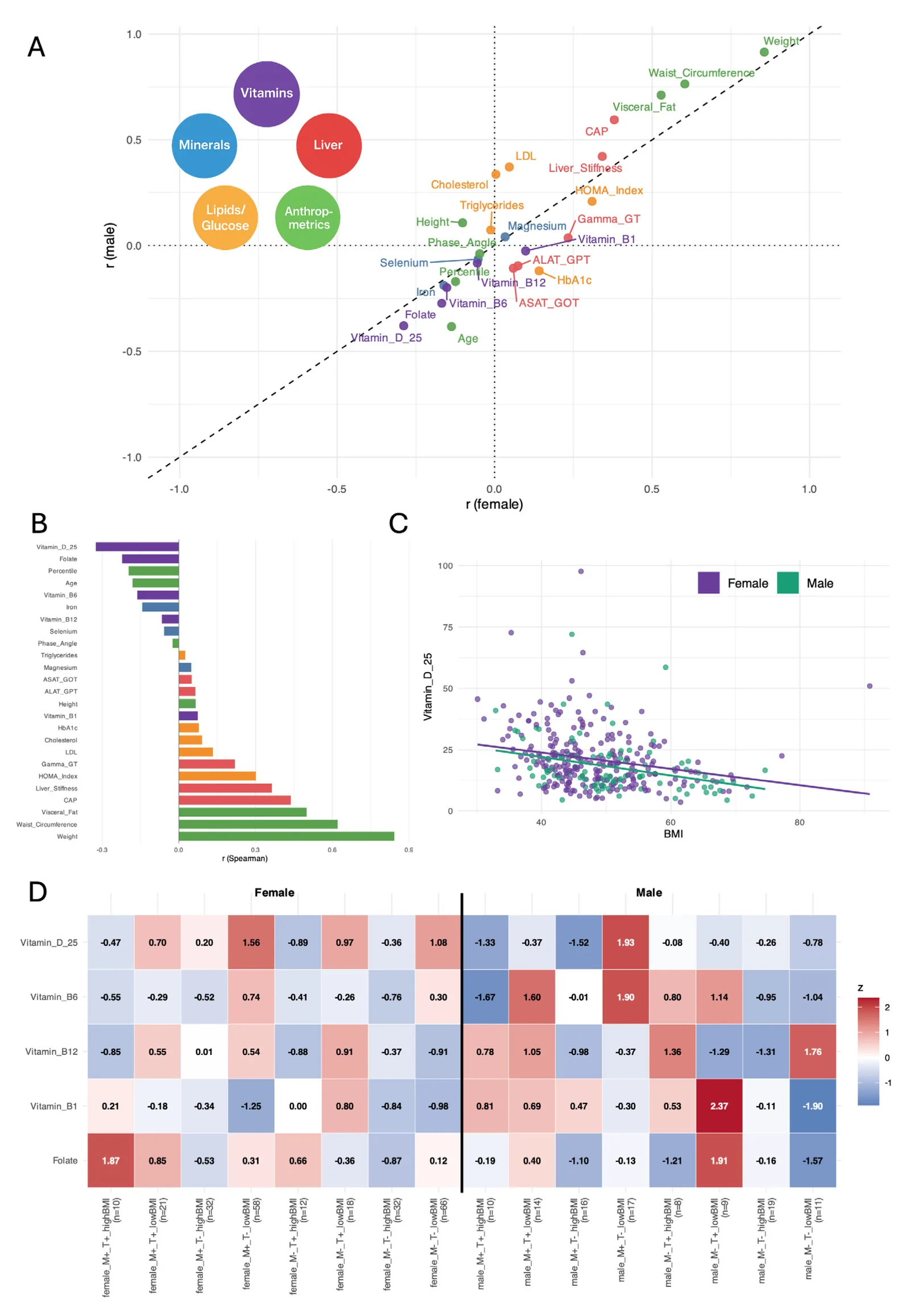
BMI–biomarker correlations and vitamin stratification. (**A**) Sex-stratified Spearman correlations of BMI with each biomarker (x = correlation in women, y = correlation in men; dashed line y = x), colored by functional family. (**B**) Pooled, sex-agnostic Spearman correlations of BMI with all biomarkers, ranked by coefficient. (**C**) BMI versus 25-OH vitamin D, shown separately for women and men with linear fits. (**D**) Exploratory heatmap of the mean concentration of five key vitamins across the 16 Sex × MASLD × T2DM × BMI-group strata, z-scored row-wise per vitamin (blue = relatively low, red = relatively high), the vertical line separates female from male strata.

To examine how adiposity relates to metabolic and nutritional status, we first computed sex-specific Spearman correlations between BMI and all quantitative variables (Figure 2A). Each point represents one variable, with female correlations on the *x*-axis and male correlations on the *y*-axis. The points aligned closely along the diagonal, indicating a high degree of concordance between women and men (Pearson r = 0.89, *p* < 10^−8^). Thus, BMI influences biochemical and anthropometric parameters in a broadly similar manner across sexes. Only a few parameters deviated modestly from the diagonal. Most strikingly, several vitamins clustered in the lower-left quadrant, signifying consistent negative associations with BMI in both women and men. Vitamin D correlated negatively with BMI in females (r = −0.289, *p* = 4.3 × 10^−6^) and males (r = −0.379, *p* = 8.4 × 10^−5^), marking it as one of the strongest shared negative relationships. Similar but weaker patterns were observed for folate (female: r = −0.168, *p* = 9.2 × 10^−3^; male: r = −0.273, *p* = 5.7 × 10^−3^) and vitamin B6 (female: r = −0.152, *p* = 0.019; male: r = −0.199, *p* = 0.046). This broad consistency suggests that higher adiposity is linked to a gradual depletion of micronutrients across both sexes, rather than sex-specific deficiencies. Encouraged by the strong cross-sex correlation structure, we next analyzed the BMI associations in the combined cohort (Figure 2B). Ranking the overall correlations revealed a continuous gradient of metabolic change with increasing BMI. Anthropometric measures dominated the positive end, as expected; weight (r = 0.842, *p* < 10^−16^) and waist circumference (r = 0.621, *p* < 10^−16^) exhibited the strongest correlations, followed by hepatic parameters such as the controlled attenuation parameter (CAP; r = 0.438, *p* = 5.5 × 10^−5^) and liver stiffness (r = 0.363, *p* = 9.9 × 10^−4^). The HOMA index as a marker of insulin resistance also increased with BMI (r = 0.301, *p* = 2.6 × 10^−6^), as did the hepatic enzyme γ-GT (r = 0.219, *p* = 3.4 × 10^−5^); triglycerides, by contrast, were not significantly correlated with BMI in this exclusively obese cohort (r = 0.025, *p* = 0.64).

At the opposite end, the strongest negative correlations again involved markers of micronutrient status rather than markers of cellular integrity such as the bioelectrical phase angle, especially vitamins. Among the micronutrients, vitamin D showed the most pronounced inverse association with BMI (r = −0.325, *p* = 5.4 × 10^−10^), followed by folate (r = −0.222, *p* = 3.4 × 10^−5^) and vitamin B6 (r = −0.163, *p* = 2.5 × 10^−3^); the body-composition percentile and age showed inverse correlations of similar magnitude (r = −0.197 and r = −0.182). These consistent inverse relationships across several vitamins highlight a broad micronutrient deficit with increasing adiposity. In contrast, bioelectrical phase angle, previously linked to muscle quality and cell mass, showed only a weak and statistically non-significant correlation (r = −0.025, *p* = 0.67) in this cohort with severe-to-extreme obesity, suggesting that electrical tissue properties may be less discriminative once BMI exceeds conventional ranges.

To visualize this relationship in detail, we plotted BMI against vitamin D levels separately for women and men (Figure 2C). Both sexes showed clear and statistically significant negative correlations with parallel regression slopes, highlighting that the decline in vitamin D is proportional and sex-independent. This trend underscores that vitamin D deficiency is a common biochemical correlate of obesity rather than a sex-specific effect. To test whether micronutrient status changes continuously with adiposity or shows a threshold at BMI ≥ 50 kg/m^2^, we compared obesity class III (BMI 40–49.9 kg/m^2^) with the extended classes IV–V (BMI ≥ 50 kg/m^2^, often termed super-obesity) for each analyte (Mann–Whitney tests with Holm adjustment) and additionally fitted segmented (break-point) regressions with a Davies test for a change in slope (Supplementary Table S3). Only folate declined significantly further in classes IV–V (median 4.2 vs. 5.9; Holm-adjusted *p* = 0.013), with vitamin D showing a non-significant trend (median 14.8 vs. 17.9; *p* = 0.074); where the Davies test indicated a change in slope (vitamins B1, B12, D and folate), the estimated break-points lay well away from 50 kg/m^2^ (range ≈ 37–66 kg/m^2^), arguing against an abrupt threshold at BMI ≥ 50 kg/m^2^ for most micronutrients. Overall, these analyses reveal a coherent, graded association between increasing adiposity and shifts in both metabolic and nutritional domains. Strong positive correlations with hepatic and anthropometric traits accompany a parallel, systematic decline in several vitamins (D, folate, and B6) and a weaker decline in iron. The near-perfect cross-sex concordance supports the conclusion that these relationships reflect fundamental metabolic features of obesity rather than secondary sex differences.

While BMI alone explained much of the observed variation in micronutrient levels, the overall similarity between men and women in simple correlation analyses suggested that additional factors might obscure sex-specific differences. To address this, we next considered two common metabolic comorbidities of obesity, type 2 diabetes mellitus (T2DM) and metabolic dysfunction-associated steatotic liver disease (MASLD), both of which can profoundly alter vitamin metabolism and storage. Participants were therefore stratified into 16 subgroups, combining sex (female or male), MASLD status (yes/no), T2DM status (yes/no), and BMI category (low or high; cutoff = 50). This stratification is exploratory: several cells are small (for example, only 20 participants combine T2DM, MASLD and a BMI ≥ 50 kg/m^2^), so the per-stratum sample sizes are provided in Supplementary Table S4. For each subgroup, the mean values of five key vitamins (vitamin D, B1, B6, B12, and folate) were computed and normalized by row-wise z-scoring to facilitate comparison across vitamins. The resulting pattern was visualized as a comprehensive heatmap summarizing sex-specific vitamin distributions across all disease and BMI combinations (Figure 2D). The heatmap immediately reveals that some subgroups exhibit a consistent multivitamin deficit, whereas others display globally elevated values across nearly all vitamins. Across all subgroup combinations, the lowest normalized vitamin concentrations clustered in the high-BMI strata, both with and without overt metabolic disease, consistent with adiposity itself being the dominant correlate of micronutrient depletion (Figure 2D). All these single strongest deviations occurred in the numerically smaller male strata, which contain only 8–19 participants each. The deepest depletions were vitamin B_6_ in high-BMI males with combined MASLD and T2DM (z = −1.67), vitamin D in high-BMI males with MASLD (z = −1.52) and vitamin B12 in high-BMI males without overt metabolic disease (z = −1.31). The single lowest value, vitamin B1 in low-BMI males without metabolic disease (z = −1.90), is the one exception to this pattern and derives from one of the smallest strata (n = 11). The most pronounced elevations were folate (z = +1.91) and vitamin B1 (z = +2.37) in low-BMI males with T2DM, and vitamin B6 (z = +1.90) and vitamin D (z = +1.93) in low-BMI males with MASLD. Because each of these strata comprises only 8–19 participants, the individual extreme values are exploratory and should not be over-interpreted. Together, these clusters reveal that certain subgroups, especially those with milder or isolated metabolic alterations, may exhibit compensatory or treatment-related micronutrient enrichment and that supplementing nutrition might blur this analysis. Overall, the pattern highlights that vitamin deficiencies concentrate in high-BMI groups, with B vitamins and vitamin D most strongly affected. Importantly, some columns in the heatmap displayed uniformly negative z-scores across all vitamins, indicating an overall micronutrient depletion in specific metabolic subtypes, whereas others were predominantly positive, suggesting relatively preserved status. While both sexes exhibited comparable global trends, the relative B vitamin pattern was, if anything, more favorable in men (higher vitamin B1, *p* = 0.042; no significant sex difference for B6 or B12), whereas folate and vitamin D were lower in men; across the whole cohort, vitamin D levels were on average higher in women than in men. These findings point to sex-specific modulation of micronutrient balance in obesity, intensified by the presence of MASLD and T2DM. These results motivate a detailed analysis on the biomarkers, especially the vitamins and liver parameters, included in the study with respect to the two considered diseases.

### 3.3. Liver Function Parameters Differ by MASLD, T2DM, and Sex

**Figure 3:**
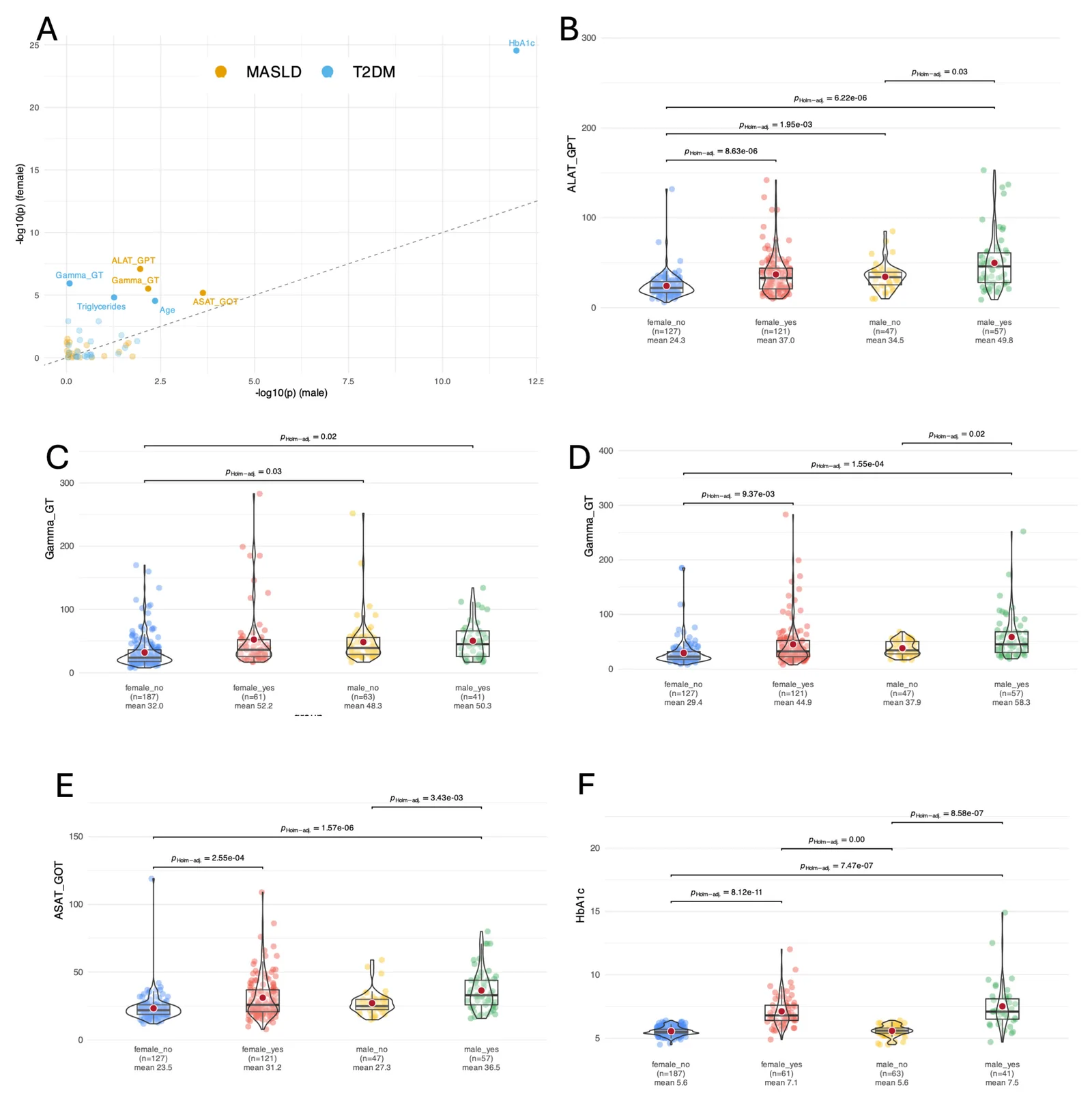
Disease- and sex-related differences in hepatic and glycemic markers. (**A**) Overview of −log10(*p*) values for the association of each biomarker with MASLD (orange) and T2DM (blue), plotted for men (*x*-axis) versus women (*y*-axis); dashed line y = x. (**B**–**F**) Group comparisons by sex and disease (individual points, group means shown as red dots; Welch ANOVA with Games–Howell post hoc tests, only significant pairwise comparisons displayed): (**B**) ALAT/GPT by MASLD; (**C**) γ-GT by T2DM; (**D**) γ-GT by MASLD; (**E**) ASAT/GOT by MASLD; (**F**) HbA1c by T2DM (*p* values displayed as 0.00 are below 0.001).

To systematically assess disease-related biochemical alterations, we next correlated all available laboratory variables with the presence of MASLD and T2DM in females and males separately. We visualized the results by plotting the negative log-transformed *p*-values for female versus male participants, thus providing an overview of parameters associated with either condition across sexes (Figure 3A). Overall, the majority of associations between MASLD (orange) and T2DM (blue) followed a similar direction in males and females, indicating largely consistent physiological effects. Notably, hepatic parameters dominated the upper right quadrant, with ALAT/GPT, ASAT/GOT, and γ-GT emerging as the strongest MASLD-associated markers, whereas HbA1c showed by far the most pronounced association with T2DM. This association is definitional rather than empirical, since HbA1c forms part of the diagnostic criterion for T2DM; the same applies to the group difference shown in Figure 3F, and it is the reason the T2DM classifier is additionally reported without HbA1c. To further explore these effects in detail, we analyzed sex-stratified group differences for the key enzymes ALAT/GPT and γ-GT. ALAT/GPT (Figure 3B) showed a robust elevation in MASLD irrespective of sex, with MASLD-positive females reaching a mean of 37.0 U/L (median 33) versus 24.3 U/L (median 22) in MASLD-negative females, and MASLD-positive males 49.8 U/L (median 46) versus 34.5 U/L (median 34) in MASLD-negative males. γ-GT, in contrast, displayed significant effects for both diabetes and liver disease. When grouped by T2DM and sex (Figure 3C), γ-GT was elevated in women with T2DM (mean 52.2 vs. 32.0 U/L), whereas in men—who had higher γ-GT overall—it differed little by diabetes status (50.3 vs. 48.3 U/L). When grouped by MASLD (Figure 3D), γ-GT followed the same pattern, with the highest values again found in males with MASLD. Aspartate aminotransferase (ASAT/GOT; Figure 3E) showed the same MASLD-associated pattern, with the highest activities in men with MASLD (mean 36.5 vs. 27.3 U/L (median 33 vs. 25) in men without MASLD, and 31.2 vs. 23.5 U/L (median 26 vs. 22) in women). Finally, HbA1c (Figure 3F) was, as expected from its role in the diagnostic definition of T2DM, markedly higher in participants with diabetes in both sexes (mean 7.51 vs. 5.58% (median 7.1 vs. 5.6) in men and 7.12 vs. 5.55% (median 6.8 vs. 5.5) in women). Together, these data confirm that both T2DM and MASLD are accompanied by distinct hepatic enzyme elevations, with males generally exhibiting higher absolute enzyme activities than females. This finding suggests potential sex-related differences in hepatic stress response or metabolic vulnerability, consistent with prior reports of sex-dimorphic liver physiology. The vitamins overall were only weakly associated with diseases, arguing for a more pronounced relation of the vitamins to BMI, as demonstrated in Figure 2. While individual clinical and biochemical variables already show distinct disease associations, such univariate correlations capture only fragments of the overall metabolic phenotype. Therefore, we sought to combine multiple markers simultaneously to evaluate their joint predictive power. By applying multivariate classification models, we aimed to determine how well the combined biochemical signature could distinguish between MASLD, T2DM, and metabolically healthy individuals.

### 3.4. Integrated Classification Identifies Distinct Biochemical Signatures for T2DM, BMI, and MASLD

**Figure 4:**
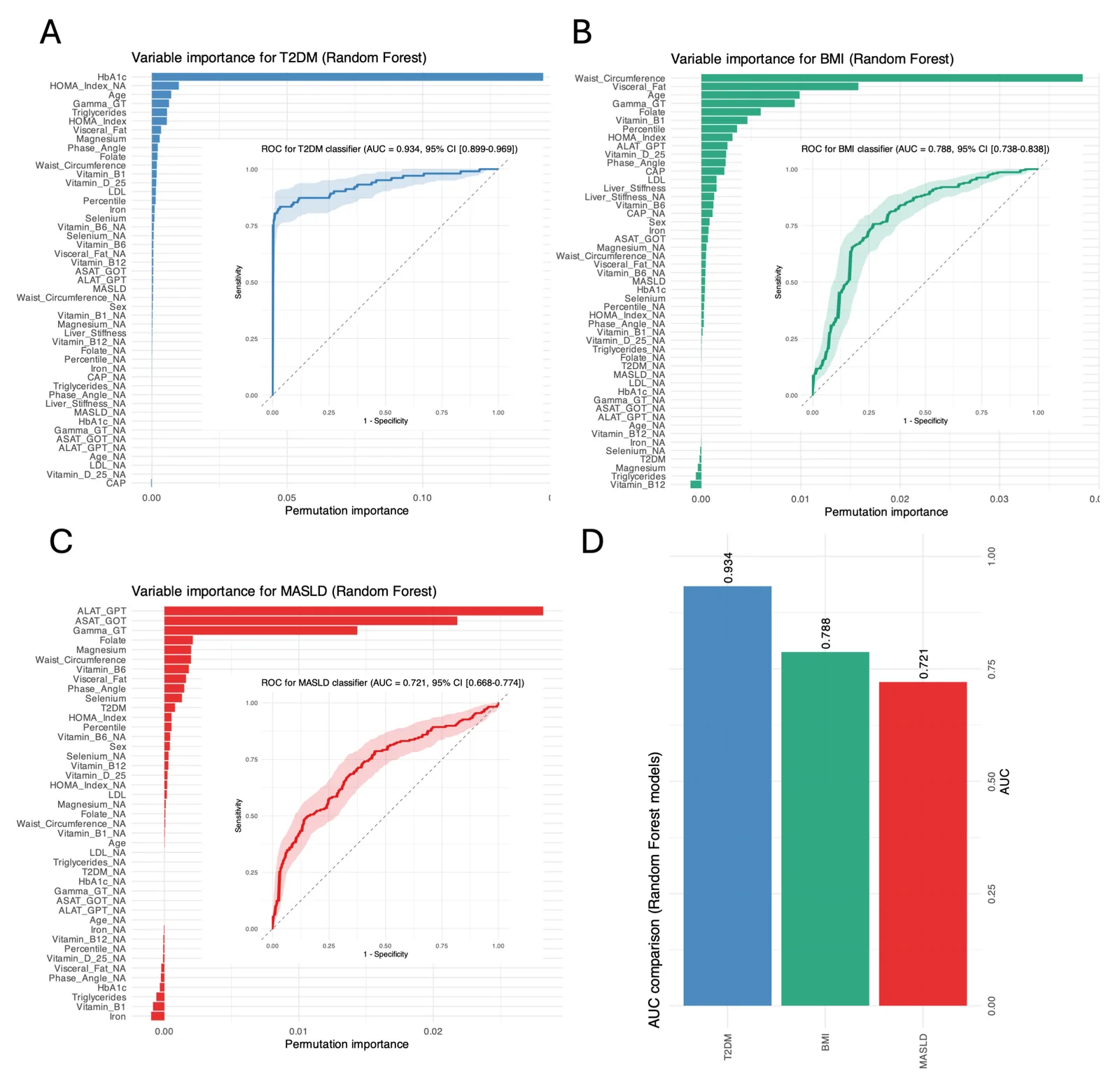
Random-forest classification of metabolic phenotypes. Receiver-operating-characteristic (ROC) curves with bootstrap 95% confidence bands for (**A**) T2DM, (**B**) high versus low BMI (cutoff 50 kg/m^2^) and (**C**) MASLD. (**D**) Comparison of the out-of-bag AUCs of the three classifiers.

To combine the information from multiple markers, we next trained random forest classifiers to predict T2DM, BMI group, and MASLD status from the complete biochemical and anthropometric dataset. Each model used identical preprocessing and parameterization to allow direct comparison of predictive performance and variable importance. The T2DM classifier achieved the highest discriminative power, with an AUC = 0.934 (95% CI 0.899–0.969) (Figure 4A). The strongest predictor by a wide margin was HbA1c (permutation importance = 0.14), consistent with its central role in the T2DM definition; it was followed, at substantially lower importance, by the missing-value indicator of the HOMA index, age, γ-GT and triglycerides, confirming the metabolic and hepatic co-activation typical of diabetic dysmetabolism. Because HbA1c is itself part of the diagnostic definition of T2DM, we repeated this model without HbA1c; discrimination remained good (AUC = 0.827, 95% CI 0.778–0.877; Supplementary Figure S3), indicating that the classifier does not merely restate its own defining criterion. All random forest models are therefore reported as exploratory classification analyses rather than validated clinical prediction tools. Of note, the missing-value indicator of the HOMA index (i.e., whether fasting insulin had been measured; HOMA index NA in Figure 4) itself showed relevant feature importance. The BMI classifier performed moderately well (AUC = 0.788, 95% CI 0.738–0.838) (Figure 4B). Here, anthropometric parameters dominated the ranking: waist circumference and visceral fat were the leading predictors, followed by age and γ-GT, reflecting the close physiological coupling of body composition with BMI. Nevertheless, several biochemical variables—particularly folate—displayed non-negligible contributions, underscoring that systemic metabolic alterations accompany adiposity even beyond direct anthropometrics. The MASLD classifier (trained, to avoid circularity, with CAP and liver stiffness excluded from the predictors) reached an AUC of 0.721 (95% CI 0.668–0.774) (Figure 4C). Consistent with hepatic involvement, ALAT/GPT, ASAT/GOT, and γ-GT emerged as the most influential variables. The MASLD model was clearly dominated by the hepatic enzymes (ALAT/GPT, ASAT/GOT, and γ-GT), of which γ-GT was also a leading predictor of T2DM, whereas glycemic and lipid markers contributed little to MASLD discrimination once the liver enzymes were available. When comparing the three models directly (Figure 4D), T2DM showed the highest predictive accuracy, while MASLD and BMI achieved similar AUCs (0.721 vs. 0.788). Beyond the shared γ-GT signal, however, the importance profiles of the three models were largely distinct: the T2DM model relied on glycemic markers, the MASLD model on hepatic enzymes, and the BMI model on body-composition measures. Together, these findings suggest that hepatic and glycemic pathophysiology are tightly interconnected, whereas obesity itself, although correlated, exhibits a somewhat distinct feature landscape dominated by anthropometric measures.

Taken together, our analyses reveal that while obesity, MASLD, and T2DM share overlapping metabolic and hepatic signatures, particularly in liver enzymes and glucose-related markers, the patterns of micronutrient depletion, most notably in vitamins D, B6, and folate, highlight distinct nutritional vulnerabilities within these disease contexts. Overall, BMI showed a markedly stronger and more consistent relationship with vitamin status than either MASLD or T2DM, underscoring that excess adiposity itself exerts a dominant influence on micronutrient balance and should be considered jointly with comorbid disease status when assessing nutritional health in severe obesity.

## 4. Discussion

In this study, we comprehensively analyzed clinical and micronutrient profiles in a cohort of patients with severe obesity, a substantial proportion of whom presented with a BMI ≥ 50 kg/m^2^ prior to bariatric surgery. By integrating biochemical, anthropometric, and metabolic parameters, we characterized associations between vitamin levels, liver function, and metabolic comorbidities including MASLD and type 2 diabetes.

Overall, our findings are largely concordant with the existing literature linking obesity to vitamin deficiencies and metabolic disturbances. Consistent with previous studies, we observed reduced concentrations of vitamin D [33,41], B6, and folate with increasing adiposity, whereas vitamin B12 showed no correlation with BMI in this cohort. These deficiencies likely reflect altered storage and metabolism of fat-soluble and water-soluble vitamins in adipose tissue. Similarly, liver-related parameters such as ALT, GGT, CAP, and liver stiffness were among the strongest discriminators for MASLD and T2DM, supporting the concept of MASLD as the hepatic manifestation of metabolic syndrome. Notably, our data extend current knowledge by covering an extreme BMI range rarely represented in previous studies. Within this range, vitamin patterns appeared more strongly correlated with BMI and hepatic markers than with discrete disease states, suggesting that micronutrient status may primarily mirror the overall metabolic burden rather than serving as a specific disease marker. Moreover, shared feature importance profiles between MASLD and T2DM underline the close metabolic interrelation of these disorders. Together, these findings highlight the complex interplay between adiposity, micronutrient availability, and metabolic dysfunction and provide a valuable dataset for exploring nutritional status in the context of severe obesity. The sex differences observed here, higher hepatic enzyme activities and CAP in men and higher vitamin D in women, are partially consistent with sex-dimorphic fat distribution and visceral adiposity, differences in hepatic enzyme physiology, and sex differences in vitamin D metabolism and binding, although these mechanisms remain hypotheses in a cross-sectional sample; sex-dependent vitamin D metabolism and the pronounced sexual dimorphism of hepatic physiology and MASLD provide plausible bases for these observations [42,43].

However, the strength of association between vitamins and BMI exceeded that observed for disease categories such as type 2 diabetes (T2DM) or metabolic-associated steatotic liver disease (MASLD). While most studies attribute micronutrient deficiencies primarily to metabolic disease mechanisms, our results suggest that the degree of adiposity itself might be a driver or consequence of vitamin depletion. This could reflect dilutional effects due to larger body fat mass, sequestration of fat-soluble vitamins in adipose tissue, or altered hepatic metabolism independent of overt disease. Such an interpretation contrasts with studies emphasizing secondary malabsorption or inflammation as the main cause of vitamin D and B deficiencies in T2DM and MASLD [44,45]. Moreover, we observed an unexpected, marked elevation of folate (z = +1.91) and vitamin B1 (z = +2.37) among low-BMI males with T2DM, whereas other vitamins decreased progressively with metabolic burden. This finding diverges from prior reports describing a uniform decline in B vitamins with increasing disease severity. Plausible explanations could involve dietary supplementation, fortification policies, or medication effects (e.g., metformin-related compensatory supplementation) or a limited group size. Even though we included 353 participants, the subgroup of those with T2DM, MASLD and BMI ≥ 50 kg/m^2^ leaves 20 individuals. Because individual dietary intake, supplement use and medication were not systematically recorded, the divergent folate and vitamin B_1_ elevations are interpreted as exploratory and possibly supplementation- or treatment-related rather than as obesity physiology; a dedicated dietary-intake and food-group assessment was not available in this retrospective cohort and is a priority for prospective studies. Diet quality is itself increasingly recognized as relevant to the development, prevention and treatment of MASLD/MAFLD [46]. Finally, the results however suggest that vitamin metabolism in severe obesity may follow nonlinear and nutrient-specific trajectories rather than a uniform depletion pattern. Overall, our results align with the general consensus that micronutrient deficiency is a hallmark of obesity, yet they challenge the notion that metabolic diseases are the sole or even primary mediators. Instead, our data suggest that severe obesity itself constitutes a distinct nutritional phenotype, with patterns of deficiency and retention that only partially overlap with those seen in diabetes or fatty liver disease. These discrepancies underline the need for prospective, mechanistically oriented studies to disentangle adiposity-driven from disease-driven effects on vitamin metabolism.

It is also important to acknowledge limitations that should be considered when interpreting our findings. As with other clinical data sets, our data contained missing values across clinical and biochemical variables, partly reflecting the real-world nature of tertiary obesity care. Although missingness was handled through median imputation and explicit modeling of missing-value indicators in the classifiers, residual bias cannot be fully excluded. In particular, we decided to keep NA variables in the classifiers to capture procedural patterns rather than biological effects, emphasizing that predictive accuracy may in part reflect clinical decision structure rather than pure physiology. Repeating the RF classification with those variables excluded yielded comparable performance on the classification outcome (BMI 0.782 (vs. 0.788), T2DM 0.925 (vs. 0.934), T2DM-no-HbA1c 0.812 (vs. 0.827), and MASLD 0.723 (vs. 0.721)). Furthermore, our cohort intentionally represents individuals with severe to extreme obesity rather than the general population. As a result, the conclusions drawn here intentionally characterize metabolic and nutritional relationships within the uppermost BMI range and cannot be generalized or extrapolated to individuals with moderate overweight or normal weight. The cross-sectional study design additionally precludes causal inference regarding whether vitamin alterations contribute to or result from metabolic disease, a topic which is widely discussed [23]. Several further caveats temper our conclusions. Liver elastography (CAP and liver stiffness) was available for only about one fifth of participants (n = 79), so that the composite MASLD definition rests on the documented clinical diagnosis for the whole cohort and is refined by measured CAP only in this subset, which may introduce differential ascertainment, while the BMI–liver associations that rely on CAP remain limited to these participants. Because a participant was labeled MASLD-positive when either a documented clinical diagnosis or a measured CAP ≥ 275 dB/m was present, a positive label is likely to be correct (favoring specificity and a high positive predictive value), whereas some true steatosis in clinically undiagnosed participants without elastography will inevitably have been missed (limited sensitivity). The composite therefore tends to under-count rather than over-count MASLD, so the reported prevalence is a conservative lower bound and any MASLD-versus-non-MASLD contrast is, if anything, biased towards the null rather than inflated. Some stratified analyses are based on very small cells (for example, only 20 participants combined T2DM, MASLD and a BMI of at least 50 kg/m^2^) so that the subgroup vitamin patterns in Figure 2D, including the folate and vitamin B1 elevations in a small male stratum with T2DM but without MASLD (n = 9), are hypothesis-generating rather than confirmatory. Because numerous correlations and group comparisons were performed across 34 variables, 2 sexes and 2 diseases without a global correction for multiple testing, borderline associations should be interpreted cautiously. Nonetheless, all BMI–micronutrient associations reported here remained significant after Benjamini–Hochberg correction (q < 0.02; Supplementary Table S5). The classifiers were evaluated only internally (out-of-bag), without an external validation cohort, and partly recapitulate their own diagnostic definitions (e.g., HbA1c for T2DM); we therefore additionally report the T2DM classifier without HbA1c and the MASLD classifier with CAP and liver stiffness excluded from the predictors (see Results), which mitigates this circularity. Finally, the cohort was markedly sex-imbalanced (249 women versus 104 men), measurements were obtained at a single time point, and dietary intake, supplement and medication use were not systematically recorded, so that intake, supplementation, altered metabolism and hemodilution cannot be fully separated as drivers of the observed micronutrient patterns. Despite these and similar caveats, the study provides a rare and comprehensive overview of metabolic, hepatic, and micronutrient interrelations in a large clinical obesity cohort.

## 5. Conclusions

By systematically integrating correlation analyses, subgroup stratification, and machine-learning-based classification, our study delineates consistent nutritional signatures across a spectrum of severe obesity that has rarely been characterized in this detail. We demonstrate that vitamin deficiencies are tightly interwoven with metabolic and hepatic alterations, extending beyond isolated biochemical changes to reflect the broader metabolic burden. This integrated analytic framework captures the multidimensional nature of micronutrient imbalance and points to a translational potential for clinical risk assessment and personalized nutritional interventions in obesity and metabolic disease that now warrants prospective evaluation. As current guidelines partially recommend routine micronutrient assessment and, where indicated, supplementation in candidates for bariatric surgery [47,48], our findings may, pending prospective validation, help to prioritize which analytes to monitor; whether correcting these deficiencies improves clinical outcomes must be established in controlled trials.

## Figures and Tables

**Table 1:** Parameter overview of the participants included in the study. (c.f. Supplementary Table S1).

Variable	Missing	Mean	Median	SD
Age	0	44.113	43	11.962
BMI	0	48.719	47.5	8.67
Height	13	169.2	168	9.064
Phase_Angle	51	5.17	5.2	0.646
Visceral_Fat	60	8.032	6.3	4.966
Waist_Circumference	60	1.354	1.34	0.169
Weight	13	140.744	134.8	30.896
Cholesterol	2	185.735	184	43.134
HOMA_Index	118	6.502	5	5.864
HbA1c	1	6.057	5.7	1.17
LDL	2	115.009	114	34.601
Triglycerides	2	151.687	128	87.249
ALAT_GPT	1	34.17	28	22.563
ASAT_GOT	1	28.753	25	13.829
CAP	274	325.608	329	55.38
Gamma_GT	1	40.554	29	34.144
Liver_Stiffness	274	9.196	6.5	7.43
Iron	3	73.123	70	27.843
Magnesium	13	0.82	0.82	0.068
Selenium	14	79.415	79.1	14.686
Folate	12	7.021	5.3	5.969
Vitamin_B1	11	79.104	77.15	19.998
Vitamin_B12	9	463.805	415	224.324
Vitamin_B6	12	9.425	6.9	8.852
Vitamin_D_25	6	20.237	17.6	11.765
High Cholesterol	0	0.623	1	0.485
FFQ	0	NA	NA	NA
MASLD	0	0.504	NA	0.501
Nicotine	36	NA	NA	NA
Patient	0	NA	NA	NA
Percentile	51	44.291	42	33.861
Sex	0	NA	NA	NA
Statins	349	0.75	1	0.5
T2DM	0	0.289	0	0.454

## Data Availability

The data presented in this study are available from the corresponding author upon reasonable request; they are not publicly available owing to privacy and ethical restrictions applying to retrospective clinical data.

## Supplementary Materials

The following supporting information can be downloaded at: https://www.mdpi.com/article/10.3390/nu18172938/s1, Supplementary Methods (Computational analysis); Supplementary Figure S1. Side-by-side boxplots of all numeric variables by sex (two-sided Wilcoxon rank-sum test), with panels ordered by *p*-value. Supplementary Figure S2. Principal-component variable loadings. Variable loadings on the first two principal components (PC1, PC2) from the principal component analysis of all numeric variables (binary variables excluded; columns with >10% missing values removed, remaining values mean-imputed and z-scaled). Each arrow represents one variable; its coordinates give the loading on PC1 (*x*-axis) and PC2 (*y*-axis), and arrows are colored by their contribution to the two-component solution. Hepatic and anthropometric markers (weight, ALAT/GPT, BMI, ASAT/GOT) load most strongly on PC1, whereas vitamin D and folate load in the opposite direction; the corresponding numerical loadings and eigenvalues are given in Supplementary Table S2. Supplementary Figure S3. Sensitivity analysis of the T2DM random-forest classifier without HbA1c. Out-of-bag receiver-operating-characteristic (ROC) curve and feature importance for the random-forest classification of type 2 diabetes for a reduced model with HbA1c removed (AUC = 0.827, 95% CI 0.778–0.877). Because HbA1c is part of the diagnostic definition of T2DM, its inclusion partly inflates discrimination; the retained performance after its removal indicates that the classifier does not merely restate its own defining criterion. Supplementary Table S1. Baseline characteristics of the cohort (n = 353) with missingness. For every variable: number of available values, percentage missing, mean ± SD and median [IQR] for the whole cohort and separately for women and men. Categorical variables (obesity class, T2DM, composite MASLD) are given as n (%). Supplementary Table S2. Principal-component loadings and eigenvalues. Eigenvalues and explained variance of PC1 and PC2, and loadings of all numeric variables on PC1 and PC2, underlying Figure 1D and Supplementary Figure S2. Supplementary Table S3. Continuum-versus-threshold analysis (obesity class III vs. extended class IV–V). For each analyte: number of participants per class (n_III, n_IVV), Mann–Whitney *p*-svalue and Holm-adjusted *p*-value, the Hodges–Lehmann location shift (diff; class IV–V minus class III), the rank-biserial effect size (r_rb) with 95% confidence interval (lo, hi), and the segmented-regression break-point estimate (±SE) with the Davies-test *p*-value for a change in slope (*p*_davies). Only folate declined significantly further beyond BMI ≥ 50 kg/m^2^ after adjustment (Holm-adjusted *p* = 0.013); where the Davies test indicated a change in slope, the estimated break-points lay well away from 50 kg/m^2^ (≈37–66 kg/m^2^). Supplementary Table S4. Per-stratum sample sizes for the 16 Sex × MASLD × T2DM × BMI-group cells shown in Figure 2D. Strata are small in places, underscoring the exploratory nature of the heatmap. Supplementary Table S5. Multiplicity-adjusted Spearman cssorrelations between BMI and each biomarker. Pooled (sex-agnostic) Spearman rank correlation coefficients (r) between BMI and every numeric variable across the whole cohort (n = 353), with two-sided *p*-values from cor.test. Because sample size differs by variable owing to missing data, the number of complete pairs (n) is given for each row. To account for multiple testing across the 25 correlations, *p*-values were adjusted by the Benjamini–Hochberg false-discovery-rate method (q_BH) and, more conservatively, by the Bonferroni method (q_Bonferroni); rows are ordered by ascending unadjusted *p*-value. All reported BMI–micronutrient associations (vitamin D, folate, vitamin B6, iron) remained significant after Benjamini–Hochberg correction (q < 0.02). Binary variables and BMI itself were excluded. Abbreviations: BMI, body mass index; FDR, false-discovery rate.
