# Supplementary material for: Vitamin (B1, B6, B12, D, and Folate) and Mineral (Iron, Selenium, and Magnesium) Status Across the Spectrum of Severe Obesity: An Integrative Multivariate Analysis of Metabolic and Nutritional Profiles
Source: Nutrients. 2026 Sep 7;18(17):2938. doi: 10.3390/nu18172938 (PMC13567054; doi:10.3390/nu18172938)
Supplement: Supplementary file 1 [file nutrients-18-02938-s001.zip › Supplementary_methods_final.pdf]

Supplementary Material

Micronutrient status across the spectrum of severe obesity — nutrients-4432429

Supplementary Methods (Computational analysis)

All analyses were performed in R version 4.3.2. Data handling used the tidyverse (dplyr, tidyr, stringr, purrr, readr), tables and cleaning used janitor; random-forest classification used ranger; ROC curves and AUCs used pROC (DeLong 95 % confidence intervals for the AUC), with ROC confidence bands obtained by non-parametric bootstrap resampling (200 replicates); principal component analysis used FactoMineR and factoextra; group-comparison plots used ggstatsplot; break-point analysis used segmented; effect sizes used effectsize; axis/colour scaling used scales.

Data pre-processing and harmonisation. The raw dataset was imported as a tab-delimited text file. Values encoded as empty strings, “NA” or “n/a” were standardised to R’s NA; decimal separators were harmonised. Continuous variables were inspected for outliers, implausible ranges and number of unique values; variables with fewer than three unique numeric values were excluded from correlation analyses. Binary variables (MASLD, T2DM, high cholesterol; statin use was documented for only 4 of 353 participants and was therefore not analysed) were retained for stratified group comparisons but excluded from correlation and PCA analyses. Each variable was assigned to one of five functional families used for colour coding throughout: Vitamins (B1, B6, B12, D-25, folate); Minerals (magnesium, selenium, iron); Liver markers (CAP, liver stiffness, ASAT/GOT, ALAT/GPT, γ-GT); Lipids/Glucose (cholesterol, triglycerides, LDL, HbA1c, HOMA1-IR); Anthropometrics (height, weight, waist circumference, visceral fat, phase angle, percentile, age). Data verification and curation. Before analysis the source records were verified and data curation was kept to the minimum necessary: one participant was excluded because of active hepatitis; six anthropometric transcription errors (height, weight) were corrected against the source records (each re-checked so that weight/height^2 reproduced the recorded BMI to within 0.1 kg/m^2); and three implausible single laboratory values (one LDL, one total cholesterol, one triglyceride) as well as two implausible height/weight pairs were set to missing. The analysed cohort comprised 353 participants. A food-frequency questionnaire flag was available for 67 participants; because an analysis restricted to these 67 individuals would be statistically underpowered and unstable, dietary-intake data were not analysed.

MASLD definition. MASLD was defined as a composite: a participant was classified MASLD-positive if either a documented clinical diagnosis of hepatic steatosis or a measured controlled attenuation parameter (CAP) ≥ 275 dB/m (where liver elastography was available) was present. Liver stiffness was analysed separately as a fibrosis marker and did not enter the MASLD definition. T2DM definition. In the same way, T2DM was defined as a composite: a participant was classified T2DM-positive if either a documented clinical diagnosis of type 2 diabetes or a measured HbA1c ≥ 6.5 % was present. Because a documented diagnosis may under-detect diabetes, this composite identified 15 participants with an HbA1c in the diabetic range but without a prior documented diagnosis as newly diagnosed cases, giving a composite T2DM prevalence of 28.9 % (102/353). The HbA1c-defining role is the reason the T2DM classifier is additionally reported without HbA1c (see below).

Correlation analyses. Spearman rank correlations were computed between BMI and every numeric variable (binaries and BMI itself excluded) using cor.test(method = "spearman"), for the whole cohort and separately in women and men. Variables were retained only with sufficient variability and sample size in both sexes (≥ 10 distinct values overall, ≥ 10 non-missing values per sex, ≥ 3 distinct values per sex). To address multiple testing, the p-values of the pooled BMI–biomarker correlations were additionally adjusted by the Benjamini–Hochberg (false-discovery-rate) and Bonferroni methods; adjusted q-values are reported in Supplementary Table S5. To test whether the BMI–micronutrient

slopes differed by sex, a BMI × sex interaction term was fitted for the key vitamins in an ordinary-least-squares model (Y ~ BMI × sex).

Principal component analysis. Numeric variables (binaries excluded) with ≤ 10 % missing values were retained; remaining missing values were mean-imputed and all variables were z-scaled before PCA. The first two components are reported; loadings and eigenvalues are given in Supplementary Table S2.

Random-forest classification. Separate random forests (ranger; 1000 trees; probability forests; permutation importance) were trained to classify T2DM, BMI group (< 50 vs ≥ 50 kg/m²) and composite MASLD from the numeric predictors. Missing predictor values were median-imputed with additional binary missing-value indicators; sex was included. Discrimination was assessed by out-of-bag ROC/AUC with DeLong 95 % confidence intervals (pROC); ROC confidence bands were derived by bootstrap resampling (200 replicates). To limit circularity, two sensitivity analyses are reported: the T2DM model was re-fitted without HbA1c (part of the T2DM definition), and the MASLD model was fitted with CAP and liver stiffness (which enter the MASLD definition) excluded from the predictors. All random-forest models are reported as exploratory classification analyses, not validated clinical prediction tools.

Continuum-versus-threshold analysis. To test whether micronutrient status changes continuously with adiposity or shows a threshold at BMI ≥ 50 kg/m², each analyte was compared between WHO obesity class III (BMI 40–49.9 kg/m²) and class IV–V (BMI ≥ 50 kg/m²) using Mann–Whitney tests with Holm adjustment and rank-biserial effect sizes, complemented by segmented (break-point) regressions with a Davies test for a change in slope (Supplementary Table S3).

Software environment. All analyses were run in R 4.3.2 on macOS. Package versions: dplyr 1.2.1; tidyr 1.3.2; readr 2.2.0; purrr 1.2.2; stringr 1.6.0; ggplot2 4.0.3; janitor 2.2.1; ranger 0.18.0; pROC 1.19.0.1; FactoMineR 2.14; factoextra 2.0.0; ggstatsplot 1.0.0; segmented 2.2.1; effectsize 1.0.2; scales 1.4.0; ggcorrplot 0.1.4.1; ggrepel 0.9.8; pheatmap 1.0.13; broom 1.0.12. Random seeds were fixed (set.seed(42)) for the random-forest and bootstrap analyses.
